# Incidental Metastatic Lung Cancer in a Patient Being Treated for Community-Acquired Pneumonia: The Case for Lung Cancer Screening in Rural America

**DOI:** 10.7759/cureus.38213

**Published:** 2023-04-27

**Authors:** Carl T Heinrich, Stephen Stabbert, Dayan Sanchez, Jayton A Lim, David E Martin, Suporn Sukpraprut-Braaten

**Affiliations:** 1 Graduate Medical Education, Unity Health, Searcy, USA; 2 Internal Medicine, Unity Health, Searcy, USA; 3 Graduate Medical Education, Kansas City University, Kansas City , USA

**Keywords:** community aquired pneumonia, uspstf, smoking and cancer, cigarette smoking, lung cancer screening, ct chest, screening test, lung cancer prevention, lung cancer, metastatic non-small cell lung cancer

## Abstract

Lung cancer is the leading cause of cancer deaths in the United States. Efforts to decrease the number of deaths over the last decade have included the publication of guidelines by the United States Preventive Services Task Force (USPSTF) recommending annual low-dose computed tomography (LDCT) scanning in patients meeting specific criteria in order to facilitate the detection and classification of potential cancers, allowing for earlier and possibly curative intervention. Unfortunately, not every patient who meets these criteria will receive LDCT surveillance due to low socioeconomic status, geographic barriers, and limited access to healthcare related to the growing shortage of primary care physicians.

We describe a case in which a patient located in a rural southeastern region of the United States presented to the emergency room with a one-week history of fevers, cough, and shortness of breath. Chest imaging revealed findings consistent with community-acquired pneumonia (CAP). He had over a 30-pack-year history of smoking cigarettes and fit the additional criteria within the USPSTF recommendations for annual LDCT scans for lung cancer screening though no screening records were found. While being treated for CAP as an inpatient, the decision was made to perform additional imaging of the patient’s left hip, as he had been having increasing pain during the hospital stay. A mass lesion was seen on computed tomography (CT) scan in the posterior acetabular roof, prompting additional imaging and biopsy, which led to findings consistent with stage IV metastatic pulmonary adenocarcinoma.

While improvements in both imaging and classification of potentially malignant pulmonary nodules and masses have been observed since the USPSTF recommendations were first released in 2013 and with the 2021 update, rural populations with high-risk patients who fit the criteria for LDCT scanning remain vulnerable to non-screening. This patient may have benefitted from annual LDCT screening for lung cancer. Encouraging primary care physicians to not only screen for current tobacco use but also to have necessary resources on hand in clinics to arrange for timely and appropriate screening appointments and follow-up visits is integral to improving the detection and early management of lung cancer. System-wide implementation of actions that may be carried out on multiple levels of care might provide both practitioners and patients with additional tools needed in a rural setting to decrease the number of lung cancer deaths.

## Introduction

Pulmonary malignancy remains the leading cause of cancer-related deaths in the United States, with over 230,000 new cases, or 12% of overall new cancer diagnoses, and 130,000 deaths, or 21% of all cancer deaths, reported in 2022 [1]. However, as surveillance and early detection through radiologic imaging modalities have continued to improve over the years, there is a growing opportunity to diagnose these cases early in their course to provide successful interventions prior to metastatic transformation. Computed tomography (CT) scanning for the discovery and monitoring of pulmonary nodules has become a reliable tool in screening the lungs for potential malignancies [2]. Further efforts have been made by the United States Preventive Services Task Force (USPSTF) and the American College of Radiology (ACR) in the last decade that have improved the ability to find and classify lung nodules [3]. Recommendations from the USPSTF published in 2013 in addition to ACR guidelines within the Lung Imaging Reporting and Data System (Lung-RADS) provide resources for both primary care and specialty physicians to initiate conversations with their patients and make informed decisions concerning the utility of screening [3]. The USPSTF criteria (updated in 2021) recommends annual lung cancer screening using LDCT in adults aged 50-80 years with a 20-pack-year cigarette smoking history and currently smoking or having quit within the last 15 years [4]. Screening may be stopped after the person has not smoked for at least 15 years or if a separate medical problem arises that significantly decreases their life expectancy or the ability or willingness to have potentially curative surgical interventions for lung cancer [4]. Notable changes to the prior recommendations published in 2013 include the expansion of the age group to include adults 50-54 years of age and the decrease of accumulated pack-years from 30 to 20, both decisions broadening the potential for discovering malignancies in the population [4].

Nevertheless, not every patient who qualifies for annual LDCT screening per the USPSTF recommendation guidelines will be screened [5]. Many reasons exist for this, including lack of access to appropriate healthcare resources and facilities due to geographic location, increasing financial burden leading to decreased utilization of healthcare, and continued shortage of primary care physicians [5]. Outside of social factors, lung cancer does not always have a uniform presentation, nor will it present in the same stage for each patient. Instances of bacterial community-acquired pneumonia (CAP) complicating the presentation or diagnosis of pulmonary cancers have been reported in the literature in various cases [6-8]. Pleural effusions can also complicate the presentation of lung cancer, especially in the presence of concomitant bacterial pneumonia or congestive heart failure [9].

Despite the typical presenting symptoms of pulmonary cancer, including cough, dyspnea, hemoptysis, fatigue, and weight loss associated with this malignancy, they are not always reported by patients, and atypical symptoms may predominate and give insight into the extent of active disease [10]. This report describes a case in a rural town (population < 25,000) in the southern U.S. in which a patient initially presenting with symptoms associated with pneumonia, who was diagnosed with and treated for CAP, was then additionally found to have stage IV metastatic pulmonary adenocarcinoma upon further diagnostic investigations.

## Case presentation

A 78-year-old Caucasian male presented to the emergency room with chief complaints of nonproductive cough, shortness of breath, and intermittent fevers of up to 102°F for several days. During the prior week, he also had increasing fatigue, mainly on exertion. He also had noticed some mild swelling in his legs. He also had body aches that had become gradually worse during this time period. He had gone to his primary care physician’s office where a chest X-ray showed signs consistent with pneumonia for which he had been started on a course of doxycycline. The patient had an adverse gastrointestinal reaction to the antibiotic and his symptoms did not improve significantly.

His past medical history included moderate chronic obstructive pulmonary disease (COPD) without a requirement for home oxygen, chronic atrial fibrillation with rapid ventricular response, benign prostatic hyperplasia, and chronic back pain associated with osteoarthritis. He had a remote history of left knee surgery after an injury but otherwise had no other major surgical procedures. The patient reported having quit smoking in 2010 though he had smoked one to two packs of cigarettes per day for at least 30 years. He drank alcohol rarely and denied any history of illicit drug use. He normally was ambulatory independently; however, in recent weeks, he had found it more difficult to walk without assistance due to weakness and pain. He had been using an assistance walker at home during this time. He was a retired member of the United States Air Force having served as a paratrooper and routinely made aerial jumps during his service. Both his mother and maternal brother had been diagnosed with lung cancer during their lifetimes. His home medications included apixaban, atenolol, alfuzosin, and inhaled tiotropium bromide. He had no known drug allergies.

On admission, he was febrile with a temperature of 102°F. His blood pressure was elevated at 150/92 and he was tachycardic at 126 beats per minute. He was started on bilevel positive airway pressure (BiPAP) in the emergency department after his oxygen saturation was measured at 76.8% with an arterial blood gas measuring his partial pressure of oxygen at 45 mmHg. His nasal cannula at three liters of oxygen flow had failed to improve it significantly. He appeared to be in mild respiratory distress but was still able to participate in conversation during the initial interview. Cardiopulmonary auscultation revealed an irregular heart rhythm consistent with atrial fibrillation, scattered inspiratory crackles most prominent over the right lower lung lobe, and scattered expiratory wheezing. He had 1+ bilateral lower extremity pitting edema without discoloration or concerning lesions of the skin.

Pertinent lab findings included an extremely elevated D-dimer at 9,333.77 ug/L (normal range: 0.01-499), a high procalcitonin at 1.32 ng/ml (normal range: 0.02-0.50), and a high brain natriuretic peptide level of 196 pg/ml (normal range: 0-135). Two blood cultures taken prior to antibiotic initiation showed preliminary results of gram-positive cocci and a sputum culture specimen was ordered but unable to be obtained. His COVID-19 and MRSA PCR swabs were both negative. His complete blood count also showed normocytic anemia with hemoglobin of 9.8 g/dl (normal range: 13.5-18.0) and a normal white blood cell count at 7.0 th/ul (normal range: 4.8-10.8).

**Figure 1:**
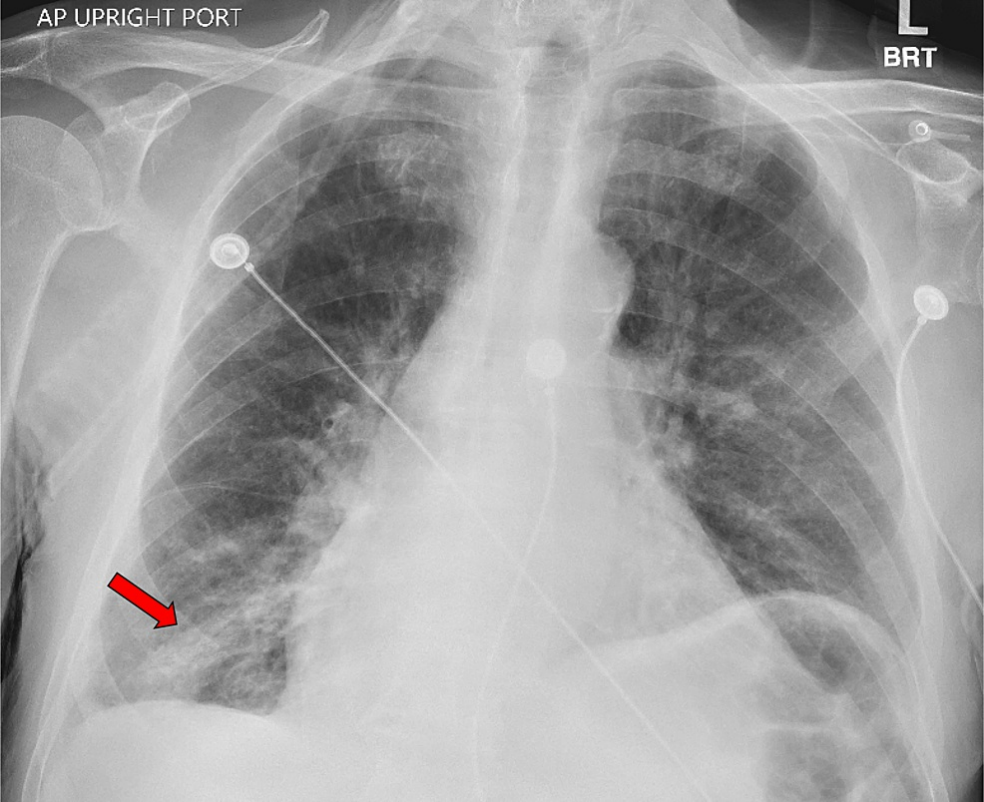
Patient’s initial AP chest X-ray taken in the emergency room A right lower lobe infiltrate can be seen (arrow) consistent with pneumonia and signs of congestive changes. AP: anteroposterior

**Figure 2:**
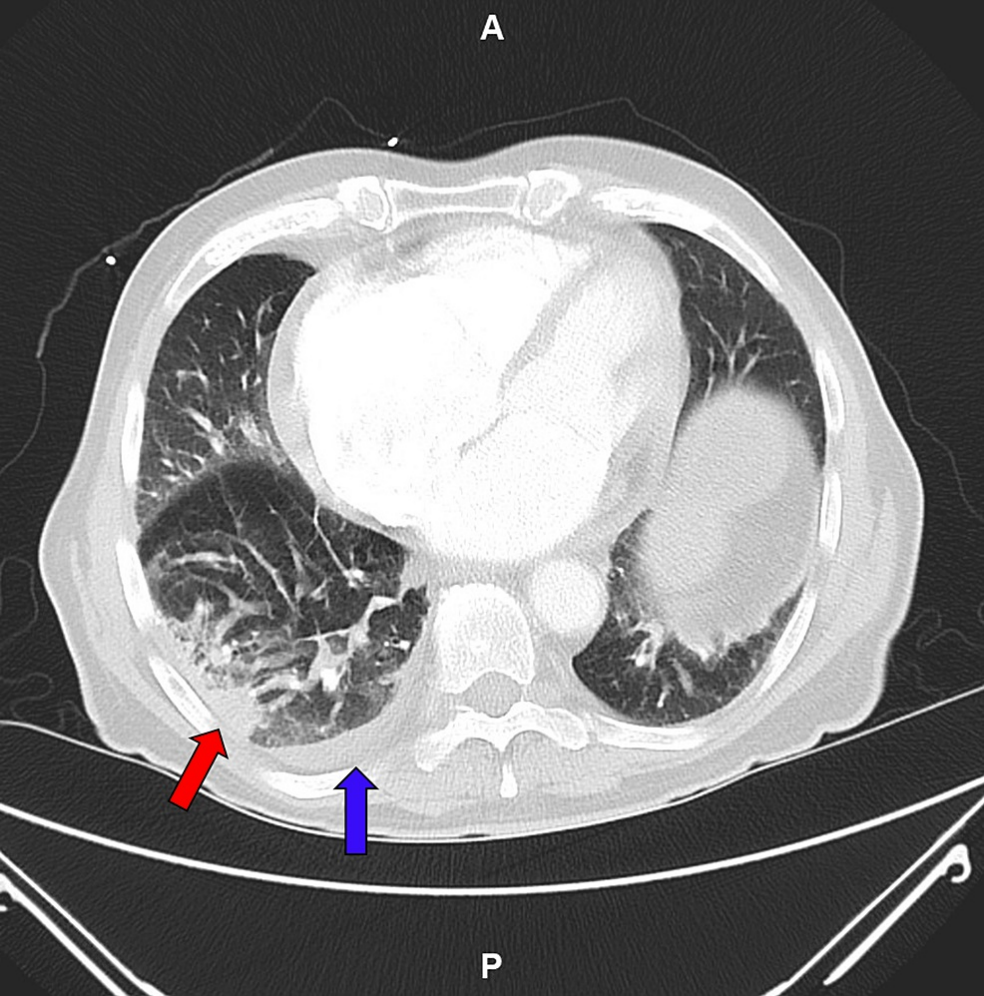
CTA pulmonary, axial view, showing a right-sided lower lung lobe consolidation (red arrow) and pleural effusion (blue), both consistent with pneumonia At this point in the hospital stay, further invasive investigation of the pleural effusion with thoracentesis was not pursued as the etiology of the fluid was presumed to be infectious. CTA: computed tomography angiogram

Imaging obtained upon admission included an electrocardiogram, chest X-ray, transthoracic echocardiogram, bilateral lower extremity venous duplex vascular ultrasound, and pulmonary computed tomography angiogram (CTA). The electrocardiogram revealed that he was in atrial fibrillation with a rapid ventricular response. The chest X-ray showed congestive changes with a right basilar infiltrate suggestive of pneumonia (Figure 1). The echocardiogram revealed bi-atrial dilation and mild concentric left ventricular hypertrophy with an ejection fraction estimated at 50-55%, indicating low-normal systolic function. The venous duplex ultrasound was negative for deep vein thrombosis. CTA pulmonary (Figure 2) revealed a right lower lobe consolidation in addition to a small right pleural effusion, bilateral hilar adenopathy, and enlarged mediastinal lymph nodes without evidence of pulmonary embolism.

After the patient was stabilized in the emergency department, he was admitted to the medical floor for treatment of CAP with acute hypoxic respiratory failure and bacteremia. It was important to rule out an additional embolic pathology based on his cardiac comorbidity, tachycardia, and hypoxia, so this, as well as beginning antibiotic treatment guided by culture sensitivities, took priority. Throughout the course of his stay, his antibiotic course included initial doses of vancomycin, ceftriaxone, and azithromycin in the emergency department, which were transitioned to cefazolin once the blood cultures returned with methicillin-sensitive *Staphylococcus aureus*. He transitioned to nasal cannula supplementary oxygen after admission with saturations measured between 92% and 96% during the stay. His home doses of apixaban and atenolol were also started for anticoagulation and rate control.

Over the following three days, the patient improved both clinically and on imaging. He felt like his ability to breathe and his cough were closer to his baseline and the subsequent chest X-ray showed signs of clearing pneumonia and decreased pleural fluid volume. The plan for discharge was to continue his intravenous antibiotic treatment via a peripherally inserted central catheter after being transferred to an inpatient physical rehabilitation facility.

**Figure 3:**
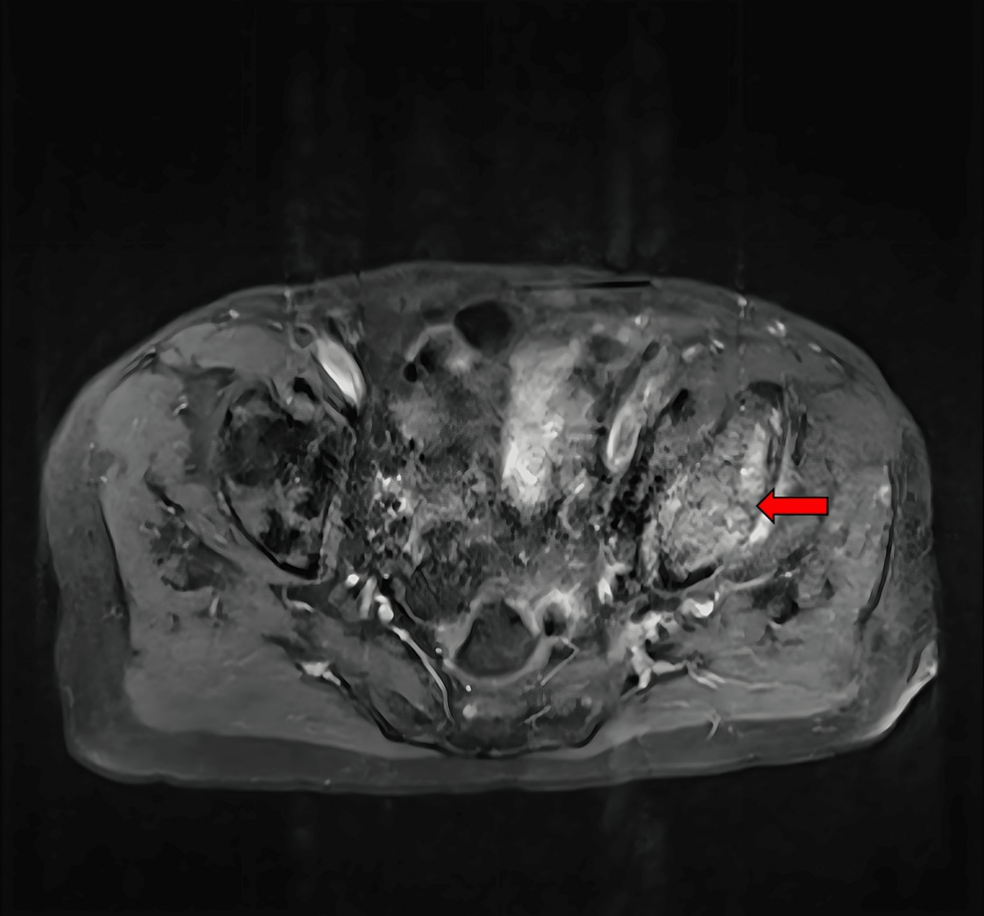
T1-weighted pelvic MRI with contrast, axial view An expansive enhancing lesion consistent in location, with the initial mass seen on a CT scan measuring greater than 6 cm in diameter, which is notably larger than originally being measurable on the CT scan, is present within the superoposterior acetabular roof of the left bony pelvis involving both the iliac and ischial segments of the hemipelvis (arrow). Cortical thinning is also present and indicates the potential for pathologic fracture with unassisted ambulation. The full image series reveals smaller enhancing lesions within the sacrum, iliac wings, and right femoral neck.

**Figure 4:**
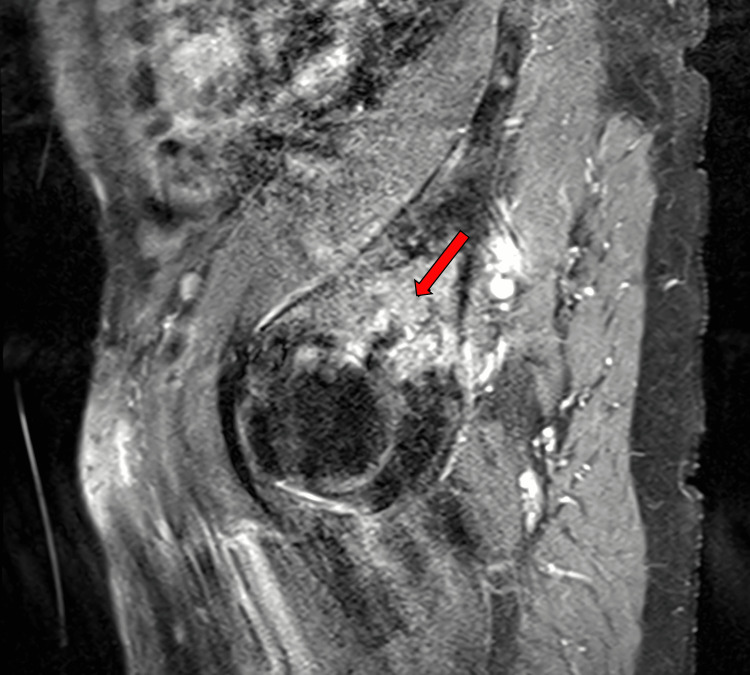
T1-weighted pelvic MRI with contrast, sagittal view An expansive enhancing lesion consistent in location, with the initial mass seen on a CT scan measuring greater than 6 cm in diameter, which is notably larger than originally able to be measured on the CT scan, is present within the superoposterior acetabular roof of the left bony pelvis involving both iliac and ischial segments of the hemipelvis (arrow). Cortical thinning is also present and indicates the potential for a pathologic fracture with unassisted ambulation. The full image series reveals smaller enhancing lesions within the sacrum, iliac wings, and right femoral neck.

**Figure 5:**
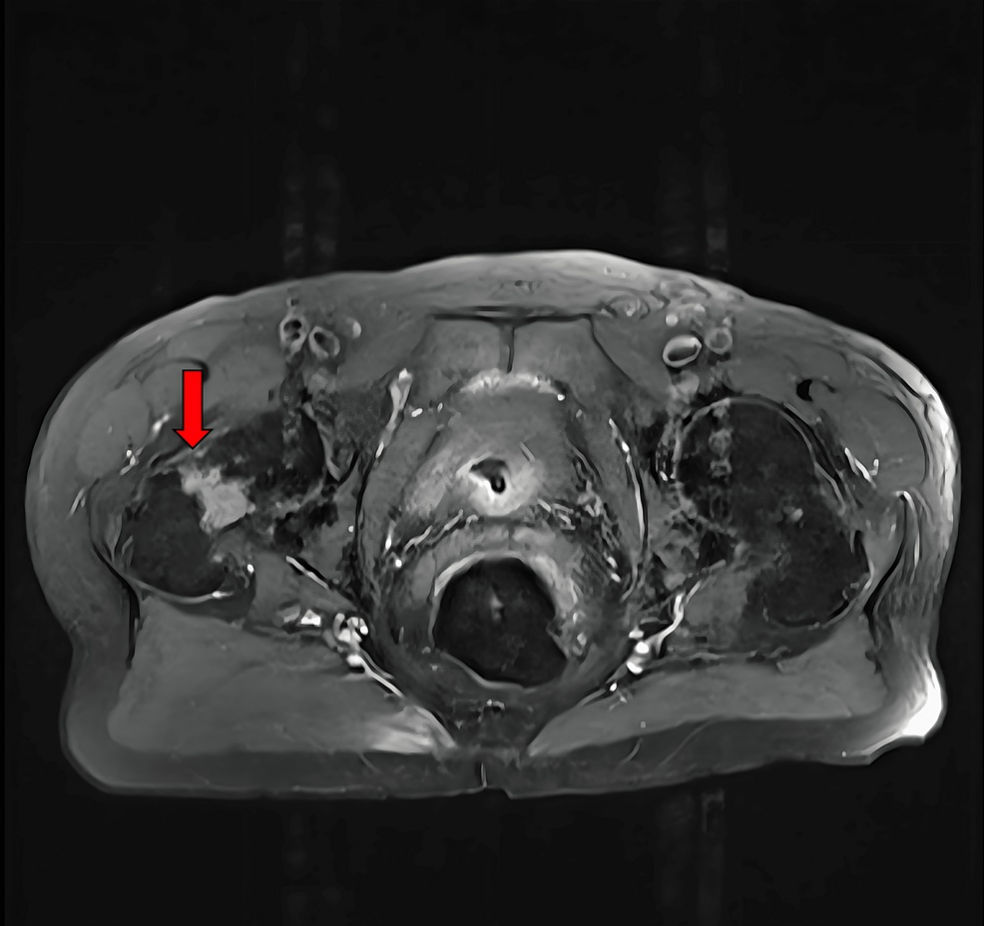
T1-weighted pelvic MRI with contrast, axial view An enhancing marrow-based lesion involving the right femoral neck is present with extension into the cortical bone (arrow). The location of this lesion indicates a high risk in this patient for a pathologic fracture with unassisted ambulation.

**Figure 6:**
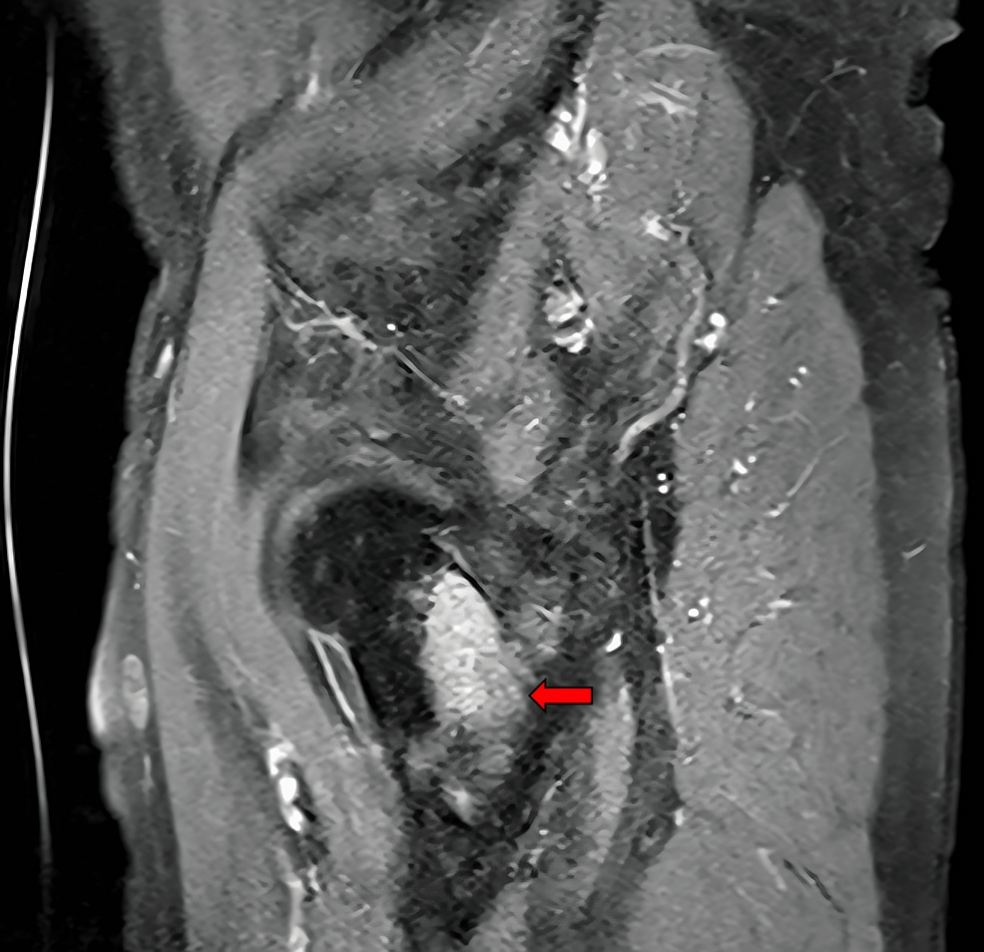
T1-weighted pelvic MRI with contrast, sagittal view An enhancing marrow-based lesion involving the right femoral neck is present with extension into the cortical bone (arrow). The location of this lesion indicates a high risk in this patient for a pathologic fracture with unassisted ambulation.

**Figure 7:**
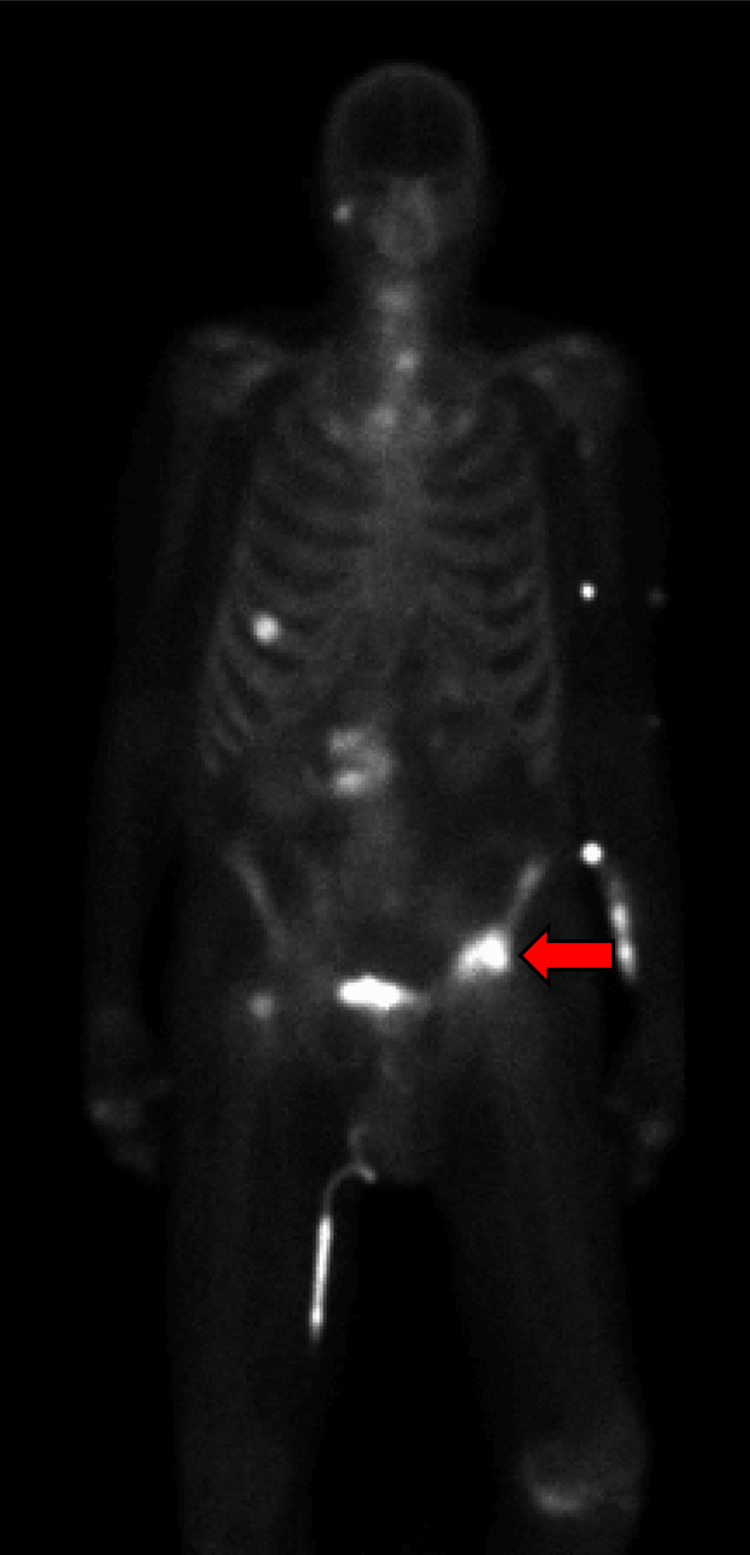
Nuclear medicine (NM) bone scan demonstrating radiotracer uptake in the right temporo-occipital skull, left proximal and mid-humerus, right eighth anterior rib, left hemipelvis, right femoral neck, sacrum, and segments of the cervical, thoracic, and lumbar vertebrae The lesion initially seen on CT and MRI in the left hemipelvis notably shows the most significant uptake among bony lesions.

During his stay, the patient brought to the primary team’s attention several times that he had been living with chronic hip and back pain, issues that he attributed to his physically demanding career in the military. His pain was controlled well with Tylenol throughout his stay, and he did not request any other medications for his pain. However, several days before the planned discharge, the patient reported that the pain in his left hip had increased during the hospital stay. He inquired if there was any imaging that could be done while he was still at the hospital prior to his transfer. He agreed to a CT scan without contrast of his left hip to investigate for any signs of acute bony pathology that revealed a mass within the left posterior acetabular roof measuring 2.7 x 3.3 x 2.6 cm in size was found. Associated thinning of the posterior cortex was also noted. Further evaluation was pursued with a pelvic magnetic resonance imaging (MRI) study that revealed enhancing bony mass lesions involving the sacrum, iliac wings, and right femoral neck (Figures 3, 4). The left acetabular lesion seen on CT was also noted to be larger than could be discerned by CT with its diameter measured at over 6 cm (Figures 5, 6) on MRI. A nuclear medicine (NM) bone scan study confirmed further masses involving the cervical, thoracic, and lumbar spine as well as the left humerus, left shoulder, right temporo-occipital region of the skull, right eighth rib, and the right medial aspect of the posterior iliac bone adjacent to the sacroiliac joint, indicating metastatic disease (Figure 7).

**Figure 8:**
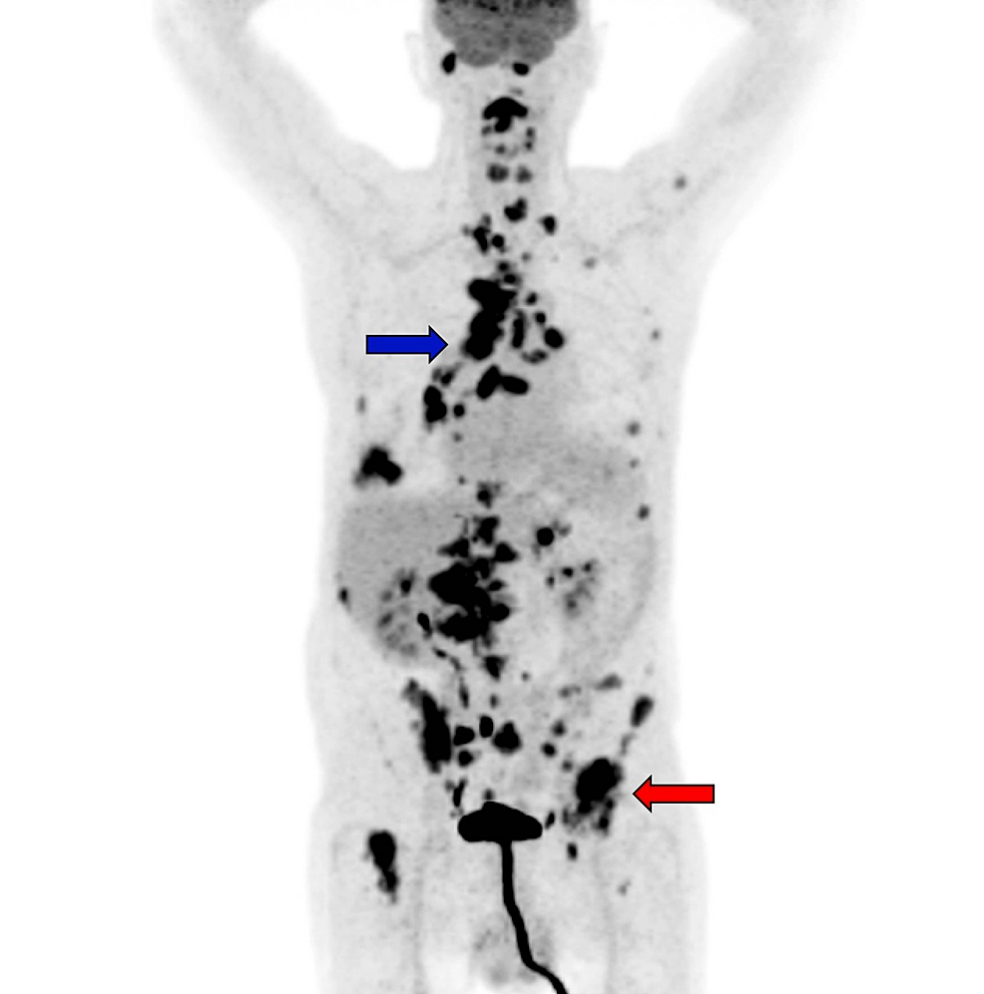
Positron emission tomography (PET) scan revealing F-18 fluorodeoxyglucose (FDG) uptake in widespread areas involving bone and soft tissue, including the right lobe of the liver, and extensive lymph node infiltration, especially within the right mediastinum and hilar regions (blue arrow). The pulmonary adenocarcinoma is suspected to have originated from the right lung.

Biopsy of the right posterior iliac lesion was performed revealing pulmonary adenocarcinoma as the primary cancer. The patient and family were counseled about the findings, and he was referred to oncology for outpatient follow-up. Further oncologic evaluation with a positron emission tomography (PET) scan revealed a metabolically active lesion in the posterior segment of the right liver lobe with the likely primary site being the right lower lung lobe (Figure 8). He was able to continue with a transfer to inpatient rehabilitation and completed therapy prior to being discharged to home with continued follow-up with his oncologist. After extensive counseling with the patient and his family about options moving forward and receiving a poor prognosis with his metastatic malignancy, the shared decision was made to move the patient to hospice home care.

## Discussion

Cases of several lung pathologies presenting simultaneously have been extensively reported in the literature. Pneumonia co-existing with pulmonary malignancy is not infrequent. One case, in particular, saw a *Klebsiella pneumoniae* infection with a right lower lobe consolidation on chest imaging continuously presenting in a patient that was ultimately found to have an underlying pulmonary malignancy [6]. The treatment team noted that while being managed as an inpatient and treated with appropriate antibiotics, the patient’s condition continued to remain stagnant [6]. Plain radiography failed to reveal signs of cancer; it was only after further investigations with a CT scan and core biopsy that primary lung adenocarcinoma was diagnosed [6]. A case of a patient diagnosed with organizing pneumonia that received further intervention with lobectomy due to a suspicious mass lesion seen on chest CT and PET scans revealed atypical epithelial proliferation on histopathological analysis [11]. At the time, the treatment team believed those changes were secondary to the infection; however, several years later, two nodules were seen along the staple line and a biopsy confirmed adenocarcinoma in both [11]. Another patient diagnosed with recurrent pneumonia who failed to improve clinically and through chest imaging with a course of oral antibiotic treatment was found to have nonmucinous bronchioloalveolar carcinoma on transbronchial biopsy of the apparent region of consolidation [7]. In a separate case, a patient who had been treated for recurrent right middle and lower lobe CAP was diagnosed with bronchoalveolar carcinoma by open lung biopsy [8]. Each of these cases suggests that when a patient who is diagnosed with an infectious pulmonary pathology fails to improve with appropriate antibiotic treatment for pneumonia, additional investigations are warranted to rule out other etiologies including malignancy [7].

Originally published in 2013 and most recently updated in 2021, the USPSTF lung cancer screening guidelines have been expanded to include more patients at an increased risk for pulmonary malignancy due to cigarette smoking history [4]. While there is no guarantee that every pre-invasive lesion will be seen on LDCT screening for lung cancer, utilization of this tool does significantly improve the likelihood that pre-invasive cancer can be found and treated [12,13]. A large meta-analysis involving 9 studies and over 96,000 subjects also found that the risks associated with false positive results, screening complications, overdiagnosis, and incidental findings were relatively low and the benefit of patients receiving appropriate screening based on the USPSTF guidelines outweighed the risks [12]. 

Underserved rural populations tend to be at a higher risk in regard to pulmonary malignancy secondary to smoking cigarettes. A 2017 study found that while disparities in smoking prevalence between urban and rural populations in the United States are likely to be multifactorial, there is still a higher prevalence of people who partake in regular cigarette smoking in rural areas compared to urban centers [14]. Innovations have been made through hospital initiatives in some rural areas in the United States and their results are being followed. One example is a rural hospital in Oregon that attempted to combat the undetected development of later-stage lung cancers and lung cancer mortality by implementing a pilot program that sought to coordinate between primary care, specialty care, and community stakeholders [15]. Emphasis was placed on identifying high-risk patients, arranging for appropriate follow-up within a reasonable timeframe, ensuring timely access to LDCT screening, and actively offering smoking cessation programs to patients [15]. Primary care providers were acknowledged as integral to all stages of the process, especially the beginning, and were involved with educational and training events throughout the duration of the initiative [15]. Internal systems and external workflow processes were developed to increase the availability of LDCT screening facilities, technology, and staff to allow timely screening for high-risk patients [15]. The primary care providers and patients then received the results with appropriate recommendations made based on radiological interpretation [15]. Finally, tailored smoking cessation support was provided to the patient through the Oregon Quit Line [15]. Between 2018 and 2020, nearly 67% of lung cancers detected through the initiative were found to be stage I or II, and the reach and adherence of the program increased annually [15]. A separate but similar initiative focused on rural regions of high tobacco usage in North Texas sought to outline logical steps to increase access to lung cancer screening resources in underserved areas [16]. This program showed that with key stakeholder involvement, reach and access can be extended to a rural population effectively [16]. A more creative approach to increase LDCT reach in rural populations involved a program that brought mobile LDCT scanning to underserved areas in the United States [17]. Initial results saw lung cancer detection rates similar to the National Lung Screening Trial and effectively reached uninsured, Medicaid, and rural patients [17].

The examples discussed provide evidence that an interdisciplinary approach involving multiple levels of care and key stakeholders in a rural, community hospital setting is capable of successfully increasing lung cancer screening reach and, by extension, early detection, to an underserved population. Continuing education of both primary care providers and specialists would also be vital in such a program, as the timely identification, referral for screening, and follow-up for high-risk patients would be essential for the program’s ability to succeed. Additional elements of tobacco cessation resources built into this type of program’s foundation would not only increase the likelihood of smoking cessation but also allow closer and more consistent follow-up for patients.

## Conclusions

We presented a case of a patient who presented with symptoms consistent with CAP and was ultimately diagnosed with stage IV metastatic lung cancer as an inpatient. Similar cases in which lung cancers were either masked by a separate pathological process or co-presented with another disease were examined and reviewed. As of 2021, the USPSTF guidelines for annual lung cancer screening with LDCT scanning have been updated to include patients between the ages of 50 and 80 (previously 55 and 80) in addition to a minimum 20 pack-year smoking history, decreased from 30 pack-years in the original 2013 recommendations. Strategies for improving screening access in rural settings were discussed as well as the importance of identifying patients at an increased risk for lung cancer meeting these criteria and arranging screening and follow-up. As lung cancer continues to be the annual leader in cancer deaths in the United States, further evaluation of the efficacy of these strategies in order to effectively apply them across a wide range of different healthcare and geographical settings is recommended.
